# The Effect of a 12-Week Health Training Program on Selected Anthropometric and Biochemical Variables in Middle-Aged Women

**DOI:** 10.1155/2017/9569513

**Published:** 2017-10-09

**Authors:** Wanda Pilch, Łukasz Tota, Ewa Sadowska-Krępa, Anna Piotrowska, Magdalena Kępińska, Tomasz Pałka, Adam Maszczyk

**Affiliations:** ^1^Department of Biochemistry and Basics of Cosmetology, Faculty of Cosmetology, University School of Physical Education in Krakow, Kraków, Poland; ^2^Department of Physiology and Biochemistry, Biomedical Science Institute, University School of Physical Education in Krakow, Kraków, Poland; ^3^Department of Physiological and Medical Sciences, Academy of Physical Education, Katowice, Poland; ^4^Department of Sports Theory, Academy of Physical Education, Katowice, Poland

## Abstract

Regular moderate physical activity positively affects health, fitness, and body composition; it regulates the pro- and anti-inflammatory cytokines levels. Vitamin D plays an important regulatory role; its adequate levels correlate with low values of inflammation markers and an increase in muscle strength and fitness in exercising people. The study's aim was to evaluate changes in somatic variables, oxidative stress, and inflammation markers, as well as blood calcidiol concentration in middle-aged healthy women after 12 weeks of aerobics classes—endurance exercises, including choreographic sequences, aiming to improve fitness and motor coordination. The training led to a significant reduction of body mass and fat tissue; it induced an increase in lean body mass. After the 12-week training program, plasma antioxidant status increased (0.65 ± 0.21, *p* < 0.01) and the concentration of lipid peroxidation products decreased (0.07 ± 0.02, *p* < 0.001). A significant increase in plasma antioxidant status associated with training could have reduced the level of proinflammatory interleukin as indicated by a positive correlation between these variables (*r*_*s*_ = 0.64, *p* < 0.05). The study proved that a 12-week health training program in physically inactive middle-aged women might provide improvements in their anthropometric parameters and selected biochemical indicators.

## 1. Introduction

Recent research has shown that regular physical activity is among the factors exerting most beneficial effects on human health. It helps maintain healthy body weight, reduces the risk of cardiovascular disease, enhances the immune system, and delays changes induced by aging [1–4]. Furthermore, regular moderate intensity physical exercise leads to the adaptive response of endogenous defence mechanisms against oxidative stress [5]. Sedentary lifestyle, on the other hand, induces overproduction of reactive oxygen/nitrogen species (RONS), responsible for the loss of muscle mass and strength as a result of an altered function of the mitochondrial respiratory chain [6].

Adequate vitamin D (D_2_ and D_3_) levels are necessary for the maintenance of structural integrity and function of the muscles [7]. Vitamin D plays an unquestionable role in bone formation and normal mineralization [8]. It stimulates calcium and phosphate absorption from the intestine and reabsorption from the kidneys, thus enhancing osteoid mineralization by calcium salts [7].

Numerous studies have confirmed vitamin D deficiency in people of different ages [9, 10]. The major circulating form of vitamin D is 25-hydroxyvitamin D (25(OH)D); therefore, the total serum 25(OH)D level is considered the best indicator of vitamin D supply to the body from cutaneous synthesis and nutritional intake. In Europe, the lowest serum 25(OH)D was found in French (25.75 ng/ml) and the highest in Spanish (34 ng/ml) women [9]. There are few studies on vitamin D levels in the Polish population, yet Napiórkowska et al. [11] revealed that 83.2% of the elderly women who participated in the study had vitamin D deficiency (<20 ng/ml).

Women with lower serum 25(OH)D exhibited higher levels of inflammatory markers than those with normal vitamin D status [12]. A strong correlation was shown between 25(OH)D levels and lipid profile changes [13, 14]. Serum 25(OH)D and physical performance in older individuals were also found to be correlated [15, 16]; vitamin D supplementation improved muscle strength in older adults [16].

In light of the aforementioned findings, it seems interesting to determine the minimal intensity of regular physical exercise that would cause beneficial changes in lipid metabolism markers and prooxidant-antioxidant balance and would reduce the inflammatory response to exercise. Hence, it was important to select a physical activity and training load that would ensure balance between reactive oxygen species (ROS) production and maintenance of muscle mass in healthy middle-aged women.

Aerobic dance exercise is popular among middle-aged women because it can help reduce depression and anxiety and improve both physiological and psychological well-being and health [17–19]. Music with slow or fast rhythm cadences, controlling and pacing the movement of selected body segments, demonstrated cardiovascular and metabolic benefits, such as increased maximal oxygen consumption (VO_2_max), improved aerobic endurance capacity, and increased energy production via the mitochondrial respiration system [20].

The presented study aimed at analyzing changes in the somatic lipid profile and prooxidant-antioxidant balance variables, as well as inflammatory markers and serum 25(OH)D levels in healthy middle-aged women after a 12-week aerobics program.

## 2. Materials and Methods

### 2.1. Characteristics of the Study Population

A total of 15 nonsmoking women (aged 42–47 years) with a low level of physical activity and similar body composition volunteered to participate in the study. Other inclusion criteria were as follows: medical report confirming lack of contraindications to physical exercise; no hypotensive, hypolipidemic, or hormonal therapy; and no antioxidant or other dietary supplements for at least 4 weeks before the training program; the subjects were also required to refrain from alcohol for at least 2 weeks prior to and during the study. The exclusion criteria were menopause and applying elimination diets (e.g., a vegan, gluten-free, or nondairy diet) for 6 months before the study or a diet resulting in nutritional deficiency as determined by the Diet 5 Software of the National Food and Nutrition Institute. The participants were asked to maintain their usual eating habits. Previous and current physical activity levels (PAL) were determined with the short version of the International Physical Activity Questionnaire (IPAQ) [21, 22], although the method is not a gold standard and allows estimations only. The study included women whose total IPAQ score was below 600 MET-minutes/week, classified as low physical activity [22].

The participants received comprehensive information on the study aims and procedures and signed an informed consent form; they were also informed that they could withdraw from the study at any time. The study was performed in accordance with the Declaration of Helsinki and approved by the Bioethics Committee of the Regional Medical Chamber (137/KBL/OIL/2013).

### 2.2. Health Training Program

The health training program, consisting of aerobics classes (high-low impact aerobics), was carried out for 12 weeks. High-low impact training is a combination of two forms of aerobics: high impact (including jumps) and low impact (in which at least one foot is always in contact with the floor). Its characteristic feature is that the jumps, typical of aerobics, are separated with marching steps. The choreographic sequence is also significant, making the movement smooth and light. The particular sets of repeated exercises had the character of endurance training and aimed to improve fitness and motor coordination. The high impact training was the most dynamic and intense type of exercise. It consisted in performing alternating jumps, leaps, running elements, and turns. The low impact training was easier for the participants and included simple sequences, marching elements, and steps.

The training sessions took place indoors, in a sports hall, between March and May, and consisted of three 45-minute meetings per week, in the afternoons, under the supervision of an instructor. During the entire training period, the subjects participated in 36 classes. All the women were equipped with heart rate monitors (Polar RS400) and maintained an individually predetermined pulse range (±4 BPM) during the classes.

During the first 6-week period, the women exercised at 70 ± 1.8% of the maximum heart rate (HRmax), with music played from CDs, with the tempo of 128–135 BPM. Within the next 6 weeks, the exercise intensity was increased to 80 ± 2.9% HRmax, and the music tempo rose to 135–145 BPM. The HRmax was estimated according to the following formula [23]:

(1) $$\begin{array}{c} HRmax=208-0.7\times age, \end{array}$$

which, for the studied group, corresponded to the value of 175 ± 4.2 BPM.

Each training session was divided into 3 parts: warm-up (5 minutes), the main part (35 minutes), and cooldown (5 minutes). The warm-up comprised a set of stretching exercises. The main workout consisted of aerobic and strength exercises performed within choreographic combinations of different intensities (high-low). The participants used their own weight to increase strength. During the first 6 weeks, 3 types of exercises were applied to enhance arm, leg, abdominal, and back muscles. Each exercise was repeated 8 times in 3 sets, which equaled 72 repetitions in all. Within the subsequent 3 weeks, the number of series was increased by 1, which resulted in 98 repetitions of the assigned exercises. In the last 3 weeks of the training program, there was again 1 series added, giving a total of 120 repetitions of particular exercises. The cooldown included relaxation exercises.

### 2.3. Anthropometric Characteristics

Anthropometric measurements were taken in the morning, after overnight fasting, before the commencement and one day after completion of the training program, in patients with an empty bladder, having washed their feet with an alcoholic disinfectant. The subjects were asked not to use creams or other foot care cosmetics within 12 hours before the measurement and to refrain from fluid intake for 4 hours before the measurements. Body height (BH), body mass (BM), fat mass (FM), fat percentage (F%), and lean body mass (LBM) were determined with bioelectrical impedance, with the use of the Jawon Medical IOI-353 body composition analyzer (Korea) in compliance with all guidelines to ensure accuracy [24]. BH was measured with a Martin anthropometer (USA) with the accuracy of 1 mm.

### 2.4. Biochemical Analyses

Fasting blood samples (6 ml) for biochemical analyses were collected into EDTA-treated vacutainer tubes in the morning, after overnight fasting, before the commencement and one day after completion of the training program. Total antioxidant status (TAS) and total oxidative capacity (TOC) of the plasma were determined with the use of commercial diagnostic kits (DM P-4100, DM P-4200, LDN Labor Diagnostika Nord GmbH & Co., Germany). Serum 25(OH)D was assessed with an immunoenzymatic test (EIA-5810, DRG Instruments GmbH, Germany). Serum cytokine concentrations (IL-6 and IL-1*β*) were measured with an immunoenzymatic test (EIA-4640, EIA-4437, DRG Instruments GmbH, Germany). Total serum cholesterol (TC), HDL-cholesterol (HDL-C), and triglycerides (TG) were evaluated by enzymatic methods using commercially available diagnostic kits (CH201, CH203, and TR210, Randox, United Kingdom). LDL-cholesterol (LDL-C) concentrations were calculated with the Friedewald formula. The risk of cardiovascular disease was evaluated on the basis of lipid ratios (TC/HDL-C, LDL-C/HDL-C, and TG/HDL-C) and the atherogenic index of plasma (AIP = log_10_ (TG/HDL), with TG and HDL-C expressed in molar concentrations) [25].

### 2.5. Statistical Methods

All statistical analyses were performed with the Statistica 10.0 software (StatSoft, Inc., Tulsa, OK, USA). The nonparametric Wilcoxon signed-rank test was used for comparisons of posttreatment data with the baseline. Spearman's rank order correlation analysis was applied to assess correlations between selected variables. The level of significance was set at *p* < 0.05.

## 3. Results

The anthropometric characteristics of the participants (Table 1) changed after the 12-week health training program. A significant reduction of the total BM (Δ = 2.4 ± 0.6; *p* < 0.001) and FM (Δ = 4.1 ± 0.9; *p* < 0.001) along with a significant increase in LBM (Δ = 1.7 ± 0.4; *p* = 0.001) was observed.

The plasma TAS, TOC, pro- and anti-inflammatory cytokines, and 25(OH)D concentrations, as well as the lipid profile, are presented in Table 2. The training program resulted in a statistically significant increase of the plasma TAS (Δ = 0.65 ± 0.21;* p* < 0.01) and a decrease in the concentration of plasma peroxidation products (Δ = –0.07 ± 0.02;* p* < 0.001). Furthermore, the training induced a significant decrease in the serum IL-1*β* (*p* < 0.05) and an increase in the serum IL-6 (*p* < 0.05). Before and after the training, the serum 25(OH)D was within the range of 24–51 ng/ml in all subjects. Importantly, the health training led to beneficial changes in the lipid profile, that is, significant reduction in LDL-C and triglycerides (*p* < 0.01 for both markers). This was associated with favorable changes in lipid ratios (TC/HDL-C and TG/HDL-C).

Spearman's rank order correlation analysis revealed statistically significant correlations between 25(OH)D levels and TAS values (*r*_*s*_ = 0.51;* p* < 0.05), triglyceride concentrations and TOC (*r*_*s*_ = 0.7;* p* < 0.05), and IL-1*β* and TAS (*r*_*s*_ = –0.64;* p* < 0.05) (Table 3).

## 4. Discussion

Physical activity has a well-established effect on body fat; independently of age, low levels of physical activity are associated with higher contents of adipose tissue [26, 27]. The regular aerobic training (high-low) performed 3 times a week for 12 weeks resulted in the reduction of BM and FM and in a significant increase in LBM. The findings regarding the beneficial effects of health training on the body's somatic components are consistent with those of other researchers [28, 29]. Nicklas et al. [30] and DiPietro et al. [31] concluded that decreases in body fat and waist circumference did not depend on training intensity. However, more recent studies brought contrary results. Greater reductions in BM, waist and hips circumference, and body mass index (BMI) and FM were found in middle-aged women participating in high intensity exercise as compared with the low intensity training group [32].

As expected, regular physical activity shifted the prooxidative/antioxidative balance towards reduction processes, which was indicated by the significantly lower plasma TOC as compared with the level before the training. The training benefits were confirmed by an increase in the participants' TAS, which remains in agreement with other authors' results [33–35]. Leelarungrayub et al. [36] observed a similar TAS increase in women aged 30–55 years after 6 weeks of moderate intensity aerobic dance exercise. Although these observations concern individuals of different ages, they seem to indicate a common mechanism of adaptation generated by regular physical activity and involving an increase in the activity of plasma antioxidative enzymes and clearance of oxidative stress products [37].

Another aim of the present study was to determine the participants' serum 25(OH)D levels. The most important role of vitamin D is to facilitate the intestinal absorption of calcium and phosphate and to increase the renal tubule reabsorption thereof. However, vitamin D also regulates a number of cell functions [7]. In the presented study, the role of vitamin D in skeletal muscle tissue [7] and its effect on white adipose tissue [38] were of particular interest. Owing to its antioxidant, anti-inflammatory, and anticancer properties, the vitamin D level is considered an indicator of overall health status. Yet, numerous researchers have found vitamin D levels beyond normal ranges. The following factors were negatively correlated with plasma 25(OH)D concentrations: female gender [39], low physical activity [35, 39, 40], indoor jobs [39], higher education and higher socioeconomic class [39], and age [41]. Our study participants, who did not take any vitamin D or calcium supplements for 4 weeks prior to the study, presented normal 25(OH)D levels. Although the 12-week training program did not have any effect on plasma 25(OH)D concentrations, the correlation analysis confirmed a statistically significant relationship between 25(OH)D levels and TAS (*r* = 0.51;* p* < 0.05). No increase was observed in plasma 25(OH)D, which was probably associated with indoor-only physical activity, and this finding is consistent with the results obtained by Scragg and Camargo Jr. [42], who concluded that regular physical activity was among the factors that increased plasma 25(OH)D levels. We did not observe a decline in vitamin D status, found by Pilch et al. [43] in women over the age of 55 who participated in a 6-week Nordic walking training program. Pilch et al. [43] noted a decrease in plasma 25(OH)D concentration, which seemed to confirm its involvement in the function of skeletal muscles.

The most recent results indicate that, along with other factors (calcium supplementation and reduction in BMI), normal 25(OH)D levels might help prevent posttraining muscle fatigue [35] by regulating the biosynthesis of several enzymes (including creatine kinase and lactic acid dehydrogenase) and troponin I. The antioxidant response is evidenced by higher TAS, which was also observed in our study. We also noted a positive correlation between 25(OH)D levels and TAS (*r*_*s*_ = 0.51;* p* < 0.05), which confirms the observations of Al-Eisa et al. [35].

Our 12-week regular physical activity program induced favorable changes in the lipid profile, consisting in statistically significant decreases in LDL-C and TG; it should be noted that the baseline lipid profile was within normal ranges. However, TC/HDL-C, TG/HDL-C, and LDL-C/HDL-C have a higher predictive value as compared with lipid profile variables [25]. The majority of research studies on the influence of various factors on the lipid panel have been performed in sedentary individuals and/or individuals with metabolic disorders. Our study participants were healthy women with normal BM, which might account for the nonsignificance of the obtained results.

Physical activity, especially regular physical activity, affects the levels of several cytokines, which, in turn, underlies metabolic adaptations and activation or quenching of the inflammatory response [44, 45]. A lot of research was performed on the kinetics of changes in cytokine levels following acute exercise [44, 46, 47] and regular physical activity [48, 49]. The effects of gender [46], training loads [50], and age [48, 49] on the kinetics of posttraining cytokine levels were also analyzed.

Among the analyzed cytokines, there was IL-6, having both pro- and anti-inflammatory properties. It stimulates the differentiation of T-lymphocytes into helper cells, able to promote B-cell activation, and, under stress (high temperature or ROS generation), induces hepatic heat shock protein expression [44, 51]. During exercise, skeletal muscles synthesize high amounts of IL-6 and release it into the blood. It was observed that long aerobic endurance exercise induced a 120-fold increase in IL-6 secretion, while short intensive anaerobic exercise decreased IL-6 production in the muscle and its release into the blood [52, 53]. Stewart et al. [54] found that muscle-derived IL-6 enhanced IL-10 and IL-1ra and inhibited the expression of IL-1*β* and TNF-*α*. The 12-week training program resulted in a slight increase in IL-6, a significant decrease in IL-1*β*, and a positive correlation between posttraining IL-1*β* and TAS (*r* = 0.643;* p* < 0.05). Zembroń-Łacny et al. [53] revealed a similar relationship between ROS biomarkers and IL-6, which indicates the contribution of ROS to IL-6 release into the blood and the antioxidant modulation of IL-6 levels. A decrease in TOC, indirectly affecting the inflammatory response, contributed to muscle tissue repair and LBM increase, as evidenced by the negative correlation between posttraining LBM and a decrease in TOC II (*r* = –0.576;* p* < 0.05). Van Hall et al. [55] also observed anabolic effects of IL-6. Our results did not confirm any statistically significant positive correlation between IL-6 and LBM.

The main limitation of our study is the lack of a control group. In our research, we evaluated the biochemical and physiological changes induced by regular training in controlled conditions in middle-aged women. Due to the fact that the results obtained before the start of the training period were considered as a reference (control values), we did not include a control group in the study.

## 5. Conclusions

The reduction of BM observed after the 12-week health training was due to the decrease in the adipose tissue level. An increase in the plasma TAS and a decrease in the plasma TOC were observed. It is possible that regular exercise reduced the level of proinflammatory IL-6, yet it had no effect on plasma 25(OH)D levels, which were within the normal range both before and after the exercise program. All these observations allowed us to conclude that the 12-week high-low aerobics health training program was beneficial for middle-aged women. It should be considered as an agent for improving the body's composition and the level of antioxidant markers and proinflammatory cytokines as well. The improvement of the lipid profile indicates a need for further investigation in a population with disturbances of cholesterol and triglyceride fractions.

## Figures and Tables

**Table 1:** The anthropometric characteristics of the studied women.

Variable	Baseline	After 12 weeks	Δ	*p*
Age (years)	42.7 ± 4.5			—
BH (cm)	165.3 ± 4.4			—
BM (kg)	65.0 ± 3.7	62.6 ± 3.9^*∗*^	−2.4 ± 0.6	<0.001
LBM (kg)	45.0 ± 4.1	46.7 ± 3.8^*∗*^	1.7 ± 0.4	<0.001
FM (kg)	20.0 ± 2.7	15.9 ± 3.3^*∗*^	−4.1 ± 0.9	<0.001
F% (%)	30.8 ± 4.2	25.4 ± 4.7^*∗*^	−5.4 ± 1.4	<0.001
BMI (kg/m^2^)	23.8 ± 2.0	23.0 ± 1.8	−0.8 ± 0.3	NS

BH: body height; BM: body mass; LBM: lean body mass; FM: fat mass; F%: fat percentage; BMI: body mass index; NS: differences not statistically significant. ^*∗*^*p* < 0.05.

**Table 2:** Metabolic variables assessed in blood samples of the study participants before and after the 12-week health training.

Measurements	Baseline	After 12 weeks	Δ	*p*
TOC (*μ*mol/l)	0.20 ± 0.04	0.13 ± 0.02^*∗*^	−0.07 ± 0.02	<0.001
TAS (*μ*mol/l)	0.80 ± 0.28	1.45 ± 0.31^*∗*^	0.65 ± 0.21	<0.01
TOC/TAS (*μ*mol/l)	0.25 ± 0.11	0.09 ± 0,09^*∗*^	−0.16 ± 0.03	<0.001
25(OH)D (ng/ml)	30.2 ± 2.6	38.4 ± 3.9	8.2 ± 2.4	0.18
TC (mmol/ml)	5.1 ± 0.9	4.8 ± 0.6^*∗*^	−0.3 ± 0.09	<0.01
HDL-C (mmol/ml)	1.7 ± 0.4	1.6 ± 0.4	−0.1 ± 0.05	1.10
LDL-C (mmol/ml)	2.9 ± 0.6	2.6 ± 0.4	−0.3 ± 0.08	<0.21
TG (mmol/ml)	1.3 ± 0.3	0.8 ± 0.3^*∗*^	−0.5 ± 0.1	<0.01
AIP (mmol/l)	−0.1 ± 0.2	−0.3 ± 0.2	−0.2 ± 0.04	0.21
TG/HDL-C	0.9 ± 0.5	0.6 ± 0.4	−0.3 ± 0.07	0.31
LDL-C/HDL-C	1.8 ± 0.5	1.7 ± 0.6	−0.1 ± 0.06	0.11
TC/HDL-C	0.9 ± 0.4	0.6 ± 0.3^*∗*^	−0.3 ± 0.1	<0.01
IL-1*β* (pg/ml)	2.56 ± 0.3	1.17 ± 0.2^*∗*^	−1.39 ± 0.5	<0.05
IL-6 (pg/ml)	36.3 ± 5.9	45.8 ± 6.5^*∗*^	9.5 ± 2.5	<0.05

TOC: total oxidative capacity; TAS: total antioxidant status; 25(OH)D: 25-hydroxyvitamin D; TC: total cholesterol; HDL-C: HDL cholesterol; LDL-C: LDL cholesterol; TG: triglycerides; AIP: atherogenic index of plasma; IL-1*β*: interleukin-1*β*; IL-6: interleukin-6. ^*∗*^*p* < 0.05.

**Table 3:** Spearman's rank correlation coefficients between selected variables.

Variables	*r*_*s*_	*p*
TAS & vitamin D	0.51	<0.05
TOC & TG	0.70	<0.05
TOC & TG/HDL-C	0.70	<0.05
TOC & AIP	0.68	<0.05
IL-1*β* & TAS	−0.64	<0.05
LBM & TOC	−0.58	<0.05
LBM & TC	−0.51	<0.05
LBM & LDL-C	−0.53	<0.05

TAS: total antioxidant status; TOC: total oxidative capacity; TG: triglycerides; HDL-C: HDL cholesterol; AIP: atherogenic index of plasma; IL-1*β*: interleukin-1*β*; LBM: lean body mass; TC: total cholesterol; LDL-C: LDL cholesterol.
